# Realizing electronically reconfigurable intrinsic chirality: from no absorption to maximal absorption of any desirable spin

**DOI:** 10.1515/nanoph-2024-0626

**Published:** 2025-02-10

**Authors:** Muhammad Ismail Khan, Tayyab Ali Khan, Moustafa Abdelbaky, Alex M. H. Wong

**Affiliations:** Department of Electrical Engineering, 53025 City University of Hong Kong, Hong Kong SAR, China; State Key Laboratory of Terahertz and Millimeter Waves, 53025 City University of Hong Kong, Hong Kong SAR, China; Department of Electrical and Computer Engineering, COMSATS University of Islamabad, Attock Campus, Islamabad, Pakistan

**Keywords:** metasurface, metamaterial, chiral, reconfigurable, circular dichroism

## Abstract

Circular dichroism – the spin-selective absorption of light – finds diverse applications in medicine, antennas and microwave devices. In this work, we propose and experimentally demonstrate an ultrathin electronically reconfigurable chiral metasurface which exploits the intrinsic symmetries of the meta-molecule to realize any spin absorption based on the handedness of the chirality chosen. We construct the left-chiral and right-chiral states by reconfiguring the meta-molecule into two enantiomeric states, which achieve strong circular dichroism exceeding 82 % at the design frequency of 9.5 GHz. The meta-molecule can be switched into a third (non-chiral) state which is isotropic and transparent. The achieved circular dichroism characteristics remain insensitive to incidence angles up to ±45°. The proposed reconfigurable chiral metasurface achieves left- and right- circular dichroism at the same frequency and with high efficiency, and is an attractive candidate for wide-ranging practical applications in imaging, wireless communication and medicine.

## 1 Introduction

Circular dichroism (CD), defined as the difference in the absorption of right-handed and left-handed circular polarizations, has attracted a great deal of attention in the scientific community due to its pivotal role in fundamental applications in chemistry, biology, pharmaceutical industry and microwave devices. CD is exhibited in several naturally occurring molecules such as proteins, DNA and viruses. The main geometrical feature giving rise to CD is the chirality of the structure. Chirality is defined as the lack of mirror symmetry: a chiral object is not superimposable on its mirror image through rotations and translations, where for two-dimensional structure the rotations are restricted to a single rotation plane while for three-dimensional structure all rotations are allowed. A chiral object and its mirror image form an enantiomeric pair: they possess the same spin-selective behavior but for opposite spins of light.

To extend the horizon of CD and overcome the limitations inherent to the natural materials such as weak and narrowband chiroptical effects, researchers turn their attentions to artificially engineered planar structures called metasurfaces which have intriguing properties of controlling the phase, amplitude and polarization of the electromagnetic (EM) waves [1], [2], [3], [4], [5]. Since it was first demonstrated that chirality can be tailored to achieve negative refractive index [5], several functional meta-devices based on chiral metasurfaces have been realized manifesting optical responses such as circular dichroism [6], [7], polarization conversion [8], [9], [10], [11], [12], [13], [14], [15] and asymmetric transmission [16], [17], [18], [19], [20]. Chiral geometries such as gammadian-shaped [21] S-shaped [22] L-shaped [23] E-shaped [24] and twisted split-rings [25], [26] have been reported to achieve chiroptical effects giving rise to spin-dependent absorption of electromagnetic waves. Both intrinsic [27], [28], [29] and extrinsic [30], [31], [32] chirality have been reported to realize spin-dependent absorption of light. Although the aforementioned designs achieve CD, they feature static meta-atoms with fixed chiral responses, which somewhat limits their spectrum of applications.

Several reconfigurable metasurfaces of varying chiral properties have been reported. In Refs. [33], [34] reconfigurable chiral metasurfaces are designed to achieve tunable asymmetric transmission of linear polarization. However, reconfigurable chiral metasurfaces with spin-selective absorption (i.e. circular dichroism) are much less explored. In Ref. [6] an Origami metasurface is claimed which exhibits spin-selective reflection and transmission, however, it is different from our work which realizes spin-selective absorption of light. Chirality tuning has been reported [7] through a phase changing material in infra-red wavelengths. However, our work is different from it as it achieves reconfigurability through electrical biasing in GHz domain for normal as well as oblique incidence which is not demonstrated for reported design in Ref. [7]. In Ref. [35] an S-shaped meta-mirror is proposed which realizes circular dichroism upon reflection in the near-infrared range. The LiNbO_3_ film is electrically biased to shift the operation frequency, but the circular dichroism is not explicitly tuned. Further, the effect has not been experimentally demonstrated. Moreover, most of the above-mentioned chiral metasurfaces operate solely upon normal incidence. A metasurface which provides full reconfigurability of the circular dichroism at the same operation frequency, and supports a wide range of illumination angles, will provide tremendous freedom in the selective tuning of circularly polarized waves, and will find use in a plethora of applications including imaging, wireless communication and medicine.

In this work, we exploit symmetries of a novel meta-molecule to realize electronically reconfigurable enantiomeric chiral states exhibiting operating-band invariant efficient spin-dependent absorption of light. Our proposed metasurface can be configured into the LC and RC states, which selectively absorb LCP and RCP light respectively. It can also be configured into a third (non-chiral) state where it is transparent in the operating band for both light spins. We switch between different states through PIN diodes which are properly positioned in the meta-molecule to demolish or construct certain symmetries depending on the on- or off-state of the corresponding diodes. We want to emphasize that switching among enantiomeric states of an intrinsically chiral meta-molecule is different from the previously reported paths to reconfigurability which switches the functionality or frequency band of operation. The former requires the fulfillment of the stringent condition to switch between two spin responses without any shift in the operating frequency band. A distinguishing feature of our design is that left- and right-chiral behaviors occur in the same operating band, which means it can be electronically configured to achieve left-handed or right-handed spin absorption for the same frequency. With this design, we realize highly efficient circular dichroism with a peak amplitude exceeding 82 % for both enantiomeric states. Moreover, the proposed design maintains its functionality against the changes in the incidence angle of the impinging wave up to 45°. The detailed experimental verification for both normal and oblique illuminations validate the functionality of the proposed metasurface.

## 2 Theoretical formulation

We begin by briefly overviewing the basic physical principles of spin dependent light–matter interactions due to which circular dichroism emerges. A basic property of light is its polarization which at the quantum level is related to the spin of the photon which can be either +1 or −1. RCP light is composed of photons with spin +1 while LCP has photons with spin −1. Linearly polarized light has photons which are in a superposition of both spin states. A non-chiral electromagnetic structure interacts with LCP and RCP light in the same manner and hence is unable to distinguish between the two spins of light. However, chiral objects can distinguish LCP and RCP light upon electromagnetic interaction. When an electromagnetic wave impinges on the surface of the metasurface, it induces electric and magnetic dipole moments in the structure which in turn generate the scattered waves. The spin-dependent light–matter interaction is intimately related with bi-anisotropy of the structure with some further constraints [3]. To enhance spin-dependent interaction, it is necessary to tailor meta-molecules to possess geometrical features enhancing bi-(an)isotropy which causes the coupling of the structure’s electric/magnetic dipole moment with the incident magnetic/electric field. Depending upon the geometrical structure of the unit cell, magnetoelectric coupling can arise between parallel components of the fields and dipole moments, such as in chiral structure helixes, or between the perpendicular components of the fields and dipole moments, as in the case of non-chiral omega particles [36]. In a chiral structure, the induced electric dipole moment produced by the co-coupling of the electric field is parallel to the induced magnetic dipole due to the cross-coupling of the electric field. Moreover, the part of the induced dipole moment caused by co-coupling (electric-electric or magnetic-magnetic) has a 90° phase difference with the part of the induced dipole moment caused by cross-coupling (electric-magnetic or magnetic-electric coupling). The chirality factor *κ* is used as a measure to quantitatively characterize bi-anisotropic electric-magnetic and magnetic-electric cross-coupling. In a particular basis system, the dipole moments and fields of a bi-anisotropic chiral medium are related by [37]:

(1) $$\left[\begin{array}{c} D\\ B \end{array}\right]=\left[\begin{array}{cc} \varepsilon & -i\kappa /c\\ i\kappa /c & \mu \end{array}\right]\left[\begin{array}{c} E\\ H \end{array}\right]$$

where ***ε***, ***μ*** and *c* are the permittivity, permeability and velocity of light while ***D*** = [*D*_*x*_, *D*_*y*_]^*T*^ is the electric displacement. The material parameters ***ε***, ***μ*** and ***κ*** are all 2 × 2 tensors. The real part of the chirality factor Re[*κ*] is responsible for the optical rotatory dispersion while the imaginary part Im[*κ*] is related with CD. The chirality factor ***κ*** causes circular birefringence: it leads to different refractive indices for RCP and LCP waves, *n*_±_ = *n*_o_ ± *κ*, leading to polarization plane rotation for a linearly polarized wave. Since the metasurface acts linearly on the incident fields and gives output fields (transmitted and reflected fields), the incident and transmitted fields are related through the following, under a particular chosen basis:

(2) $$\left[\begin{array}{c} E_{tx}\\ E_{ty} \end{array}\right]=T_{\text{lin}}\left[\begin{array}{c} E_{ix}\\ E_{iy} \end{array}\right]=\left[\begin{array}{cc} t_{xx} & t_{xy}\\ t_{yx} & t_{yy} \end{array}\right]\left[\begin{array}{c} E_{ix}\\ E_{iy} \end{array}\right]$$

where *t*_*ji*_ represents transmission coefficient when the incident linear polarization is ‘*i*’ while the transmitted field polarization is ‘*j*’. Once the transmission coefficients of the metasurface are found in a particular basis system, they be translated to any other basis, rotated with respect to original by an arbitrary angle, through suitable transformation matrices. Also, the given transmission matrix can be used to obtain transmission coefficients for the cases where the metasurface is rotated by an arbitrary angle in the same plane. Similarly, one can perform a co-ordinate transformation to find the transmission coefficients for circularly polarized waves from those for linearly polarized waves:

(3) $$\begin{array}{ll} T_{\text{cir}} & =\left[\begin{array}{cc} t_{++} & t_{+-}\\ t_{-+} & t_{--} \end{array}\right]\\ & =\frac{1}{2}\left[\begin{array}{cc} t_{xx}+t_{yy}+i\left(t_{xy}-t_{yx}\right) & t_{xx}-t_{yy}-i\left(t_{xy}+t_{yx}\right)\\ t_{xx}-t_{yy}+i\left(t_{xy}+t_{yx}\right) & t_{xx}+t_{yy}-i\left(t_{xy}-t_{yx}\right) \end{array}\right] \end{array}$$

The + symbol is used to represent RCP while ‘−’ represents LCP wave. In the same way, reflection coefficient matrix for the circular polarization, ***R***_cir_ can be obtained which contains the co- and cross polarized coefficients when the incident wave is circularly polarized. The power absorbed by the metasurface for a particular incident polarization ‘*i*’ is obtained by subtracting the transmitted and reflected power from the normalized incident power, and is given by:

(4) $$\begin{array}{c} A_{+}=1-\left({\left|t_{++}\right|}^{2}+{\left|t_{-+}\right|}^{2}+{\left|r_{++}\right|}^{2}+{\left|r_{-+}\right|}^{2}\right)\\ A_{-}=1-\left({\left|t_{+-}\right|}^{2}+{\left|t_{--}\right|}^{2}+{\left|r_{+-}\right|}^{2}+{\left|r_{--}\right|}^{2}\right) \end{array}$$

where *A*_+_ and *A*_−_ are the absorptions for RCP and LCP waves, respectively. Circular dichroism is quantitatively defined as the difference of the two absorptions:

(5) $$CD=A_{+}-A_{-}$$

Thus, a passive surface should have a CD ranging from 1 to −1: the former belonging to a surface which strongly absorbs RCP waves and transmits LCP waves without loss, and the latter belonging to a surface which strongly absorbs LCP waves and passes RCP waves without loss. The simultaneous achievement of both, at the same frequency upon proper reconfiguration, is an unprecedented feat and should have tremendous value in the control of circularly polarized waves.

**Figure 1:**
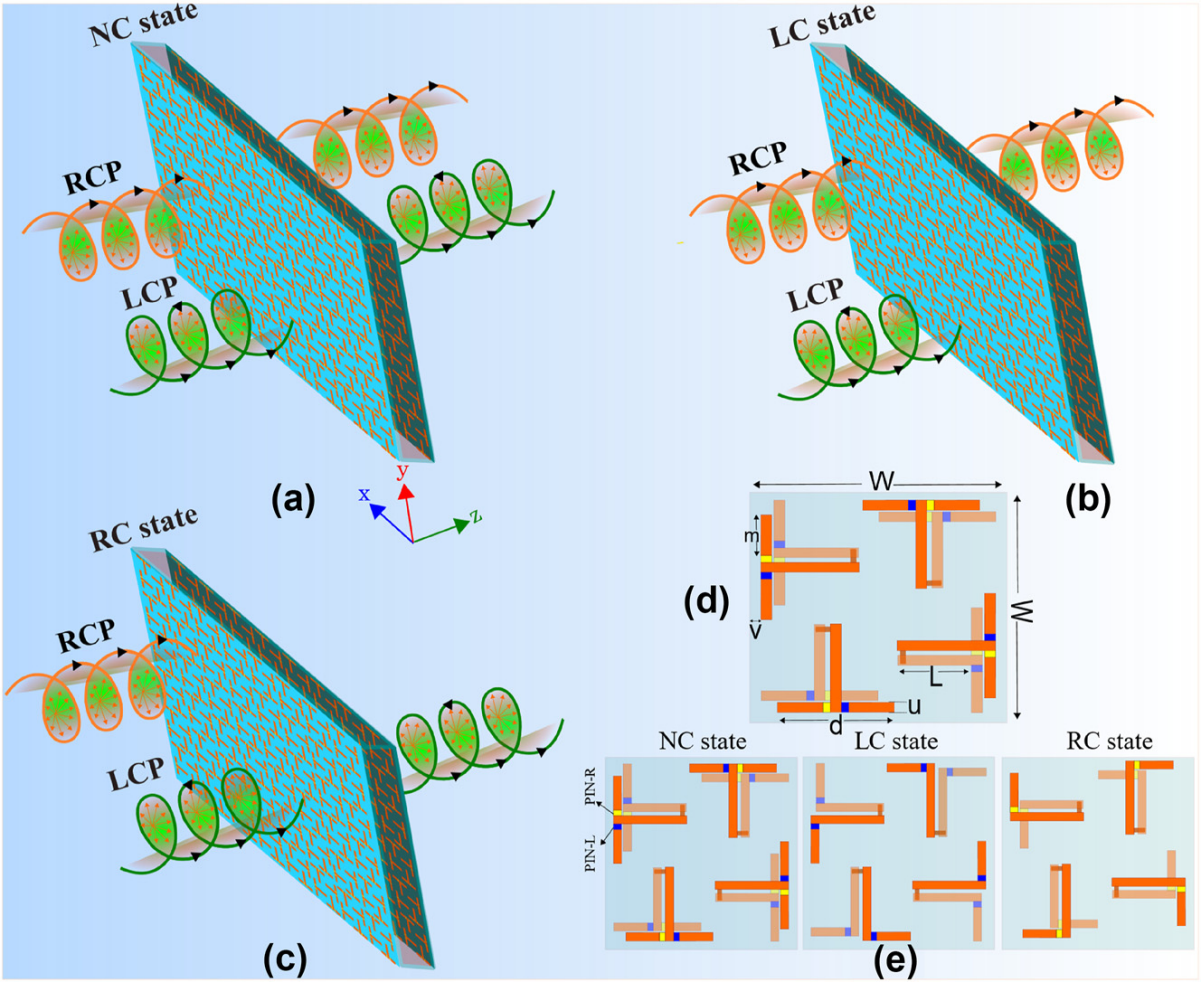
Schematic of the metasurface, (a) the non-chiral state where metasurface is transparent to both spins, (b) the left-chiral state where only left-handed spin is absorbed, (c) the right-chiral state where only right-handed spin is absorbed, (d) a single meta-molecule of the structure, (e) single effective meta-molecule in different states where for RC-state PIN-R is on and PIN-L is off while for LC-state PIN-R is off and PIN-L is on. For NC-state all PIN diodes are turned on.

Our proposed reconfigurable chiral metasurface, which accomplishes the aforementioned reconfigurable chirality, is depicted in Figure 1. The “meta-molecule” of the metasurface, as shown in Figure 1(d), is composed of four mutually rotated (by 90°) T-shaped metallic structures which we call “meta-atoms”. The meta-atoms are patterned on both sides of the dielectric spacer and connected through metallic vias. Each meta-atom contains four PIN diodes, shown in blue (PIN-L) and yellow (PIN-R), which are judiciously positioned on both sides of each T which can be independently turned on and off. By turning on and off the appropriate PIN diodes, the T-shaped scatterer, which is non-chiral, is reconfigured into a connected L-shaped scatterer, which is chiral, plus two non-resonant short metal strips which do not interact appreciably with the incident light. Based on the combination of switched-on and switched-off diodes, the meta-molecule can be switched to the left-chiral (PIN-L is on and PIN-R is off) and right-chiral (PIN-L is off and PIN-R is on) geometries as depicted in Figure 1(e). It can be noted from the two chiral states shown in Figure 1(e) that each top and bottom layer lack reflection symmetry along any axis and hence are chiral. The chirality of the complete three-dimensional meta-molecule can be verified from the lack of reflection and center inversion symmetry. Moreover, the meta-molecule has C_4_ symmetry (90° rotational symmetry) in all three states, qualifying it for an isotropic chiral structure when operated in chiral mode. It can be also observed from Figure 1(e) that the left-chiral and right-chiral meta-molecules are enantiomers as they are mirror images of each other. In the third state, all the diodes are turned on, rendering the meta-molecule mirror symmetric and hence non-chiral. The unique advantage of the designed meta-molecule is that it offers enough degrees of freedom to be tuned among two enantiomeric states (left-chiral and right-chiral) and a non-chiral state. Moreover, all achieved states possess C_4_ symmetry, which enables the metasurface to attain strong spin-selectivity for the chiral states and polarization insensitivity for the non-chiral state.

## 3 Design and simulation

**Figure 2:**
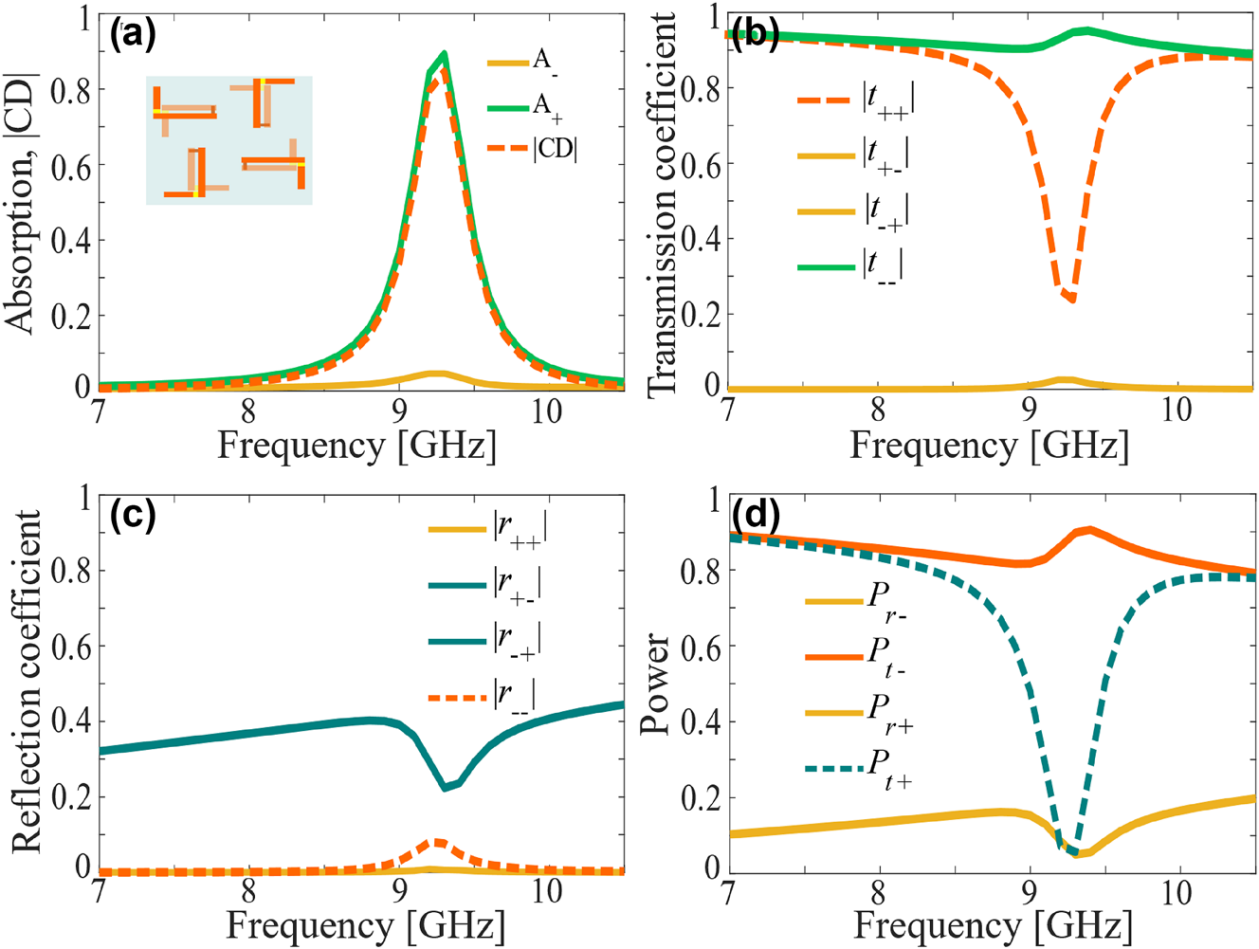
Simulation results for the RC-state. (a) Absorption and circular dichroism. (b) Magnitudes of co- and cross-polarized transmission coefficients. (c) Magnitudes of co- and cross-polarized reflection coefficients. (d) Power transmitted and reflected for LCP and RCP incidence. The inset in (a) shows the effective meta-molecule for the RC state.

We design the metasurface at the microwave regime and investigate its chiroptical response using the full-wave simulation software Ansys HFSS. The dielectric spacer is an F4B substrate, with a relative permittivity of 2.65, a loss tangent of 0.01 and a thickness of 0.8 mm, which is 0.025*λ*_o_ at the design frequency of 9.5 GHz. The metallic patterns on both sides of the spacer are made of copper with a conductivity of 5.8 × 10^7^ S/m both in simulations and experiments. The meta-molecule is repeated in the *x*- and *y*-directions using periodic boundaries. The parametrically optimized geometrical parameters of the meta-molecule, as labelled in Figure 1(d), are (in mm): *W* = 10, *L* = 3.5, *u* = 0.5, *v* = 0.5, *d* = 4.0 and *m* = 1.35. In our simulation model we use commercially available PIN diodes (MADP-000907-14020x) with RLC boundaries implementing their on- and off-state equivalent circuits. We use Floquet ports to excite the metasurface and measure the *x*- and *y*-polarized transmission coefficient matrix ***T***_lin_ introduced in (2). Thereafter, we obtain the circularly polarized transmission coefficients ***T***_cir_ through (3) and find the spin-specific absorption efficiencies (*A*_+_ and *A*_−_) and the circular dichroism (CD) using (4) and (5). Figure 2 shows the simulated performance of the metasurface when operated in the RC state. It can be seen from Figure 2(a) that in the RC state, the proposed structure absorbs RCP light with more than 87 % absorption efficiency around the resonance frequency of 9.5 GHz whereas for the LCP wave the absorption efficiency is negligibly small (less than 5 % for all frequencies). Thus, a strong CD, represented by |Δ*A*| in the figure, exceeding 85 %, is manifested around the resonance frequency in the RC state. The LCP wave is mostly transmitted with the co-polarized transmission coefficient magnitude: $\left|t_{--}\right|$ exceeds 95 % around the operating frequency as shown in Figure 2(b). This high LCP transmission efficiency is achieved across the entire frequency range because no current is induced on the right-handed chiral structure, thus allowing the wave to pass without any interaction with the metasurface. Correspondingly, as shown in Figure 2(c), the reflection coefficient magnitude within the operating band 9.3–9.6 GHz is less than 0.27, indicating that negligible power is reflected both for RCP and LCP waves. Figure 2(d) shows that across a wide bandwidth from 7 to 10.5 GHz, the incident power is transmitted with efficiency higher than 80 % for both LCP and RCP waves, with the exception that the RCP wave is absorbed strongly within the operating band. As a linearly polarized wave is a combination of RCP and LCP waves, therefore, the RC-state also acts as a linear-to-circular polarizer in the operating band by absorbing the RCP part while passing the LCP. It is also important to note that the geometrical structure of the proposed metasurface allows a symmetric response in both forward and backward direction. In other words, it absorbs the same spin both in the forward and backward direction, therefore, its response is invariant to whether the impinging wave strikes the metasurface in the *+z* or −*z* direction. Moreover, the response of the metasurface is invariant to the rotation of the metasurface by any angle in the *xy*-plane. This is attributed to the C_4_ symmetry of the meta-molecule.

**Figure 3:**
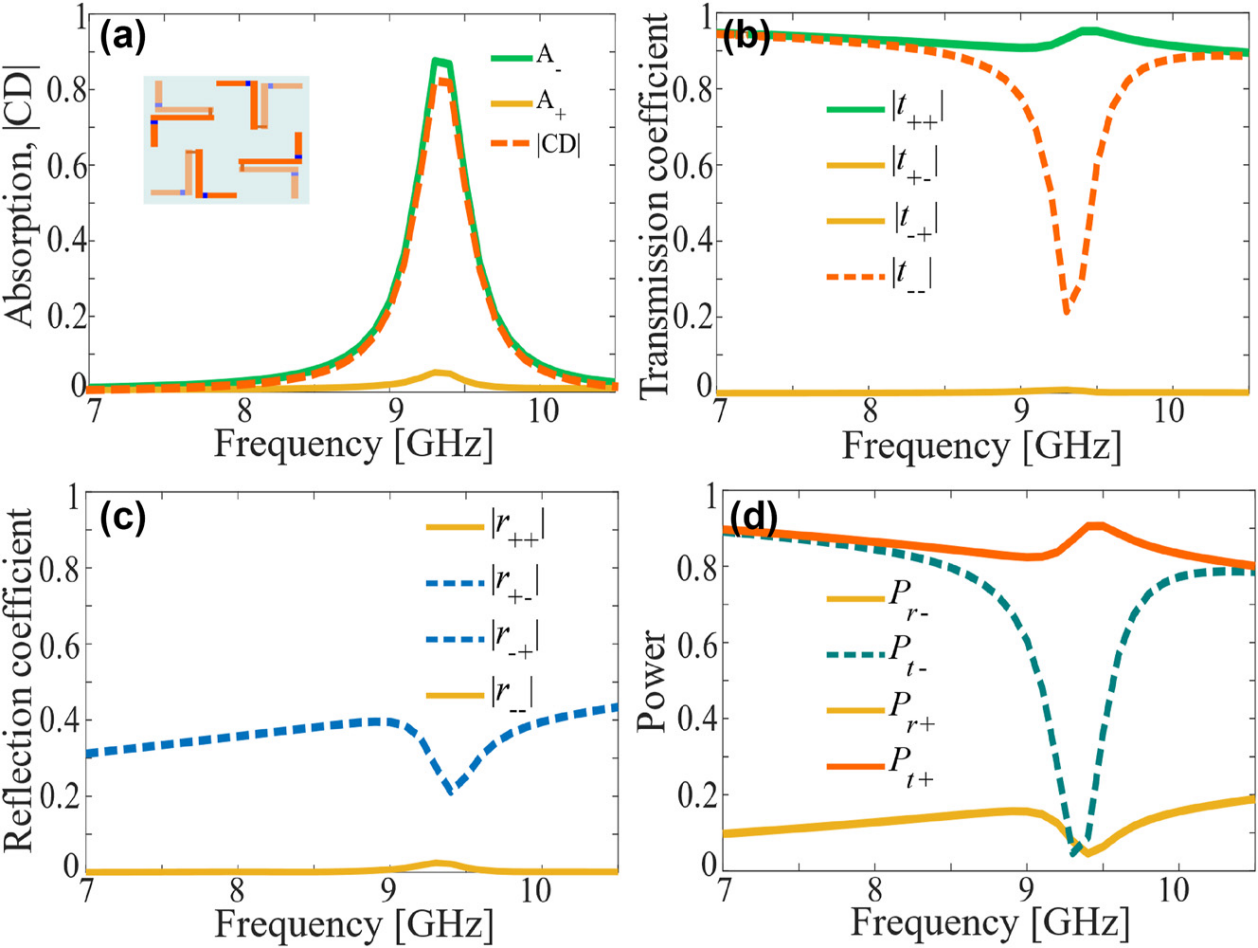
Simulation results for the LC-state. (a) Absorption and circular dichroism. (b) Magnitude of co- and cross-polarized transmission coefficients. (c) Magnitude of co- and cross-polarized reflection coefficients. (d) Power reflected and transmitted for RCP and LCP incidence. The inset in (a) shows the effective meta-molecule for the LC state.

Figure 3 shows the simulated performance of the metasurface when operated in the LC state. When the metasurface is reconfigured to the LC state, which is the mirror image or enantiomer of the RC state, its interaction with the spin of the incident wave is reversed as expected from the geometrical symmetry of the meta-molecule. Figure 3(a) show that the metasurface absorbs LCP waves with more than 87 % absorption efficiency in the operating band while the RCP wave is transmitted with less than 5 % absorption for all frequencies. Similarly, Figure 3(b) and (c) present the magnitudes of the co- and cross-polarized transmission and reflection coefficients respectively for both RCP and LCP incidence, showing that the reflection is negligible in all cases, the transmission of RCP waves is near unity ( $\left|t_{++}\right|\approx 1$ ), and the transmission of LCP waves for frequencies away from the operation band. Similarly, it can be observed from Figure 3(d) that the incident power is transmitted with efficiency higher than 90 % for both spins except in the operating band where LCP is absorbed. This time, high RCP transmission efficiency is achieved because no current is induced on the left-handed chiral structure, thus allowing the wave to pass without any interaction with the metasurface.

**Figure 4:**
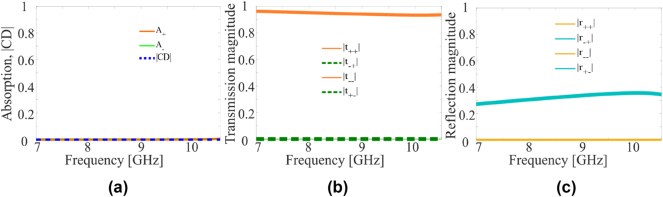
Simulation results for non-chiral state. (a) Absorption and circular dichroism. (b) Magnitudes of co- and cross-polarized transmission coefficients. (c) Magnitudes of co- and cross-polarized reflection coefficients for non-chiral state. The inset in (a) shows effective meta-molecule for the non-chiral state.

Figure 4 shows the simulated metasurface performance in the non-chiral state. In this state, all the PIN diodes are turned on resulting in a non-chiral metasurface with 90° rotational symmetry. Since the meta-molecule is non-chiral, it interacts equally with both RCP and LCP waves, leading to negligible circular dichroism as is evident from Figure 4(a). Further, since the T-shaped element is non-resonant at the operation frequency, the metasurface interacts with neither RCP nor LCP waves, and thus transmits them with high efficiency. This fact is supported by the simulated transmission and reflection spectra shown in Figure 4(b) and (c) respectively. It is also important to note that all parameters: absorption, transmission and reflection coefficients are same for both LCP and RCP waves due to the non-chiral and isotropic geometry of the structure. Moreover, the non-chiral state is symmetric along *z*-axis which results in same transmission and reflection coefficients for both forward and backward incidence. Unlike the two chiral states, the non-chiral state preserves time-reversal symmetry.

**Figure 5:**
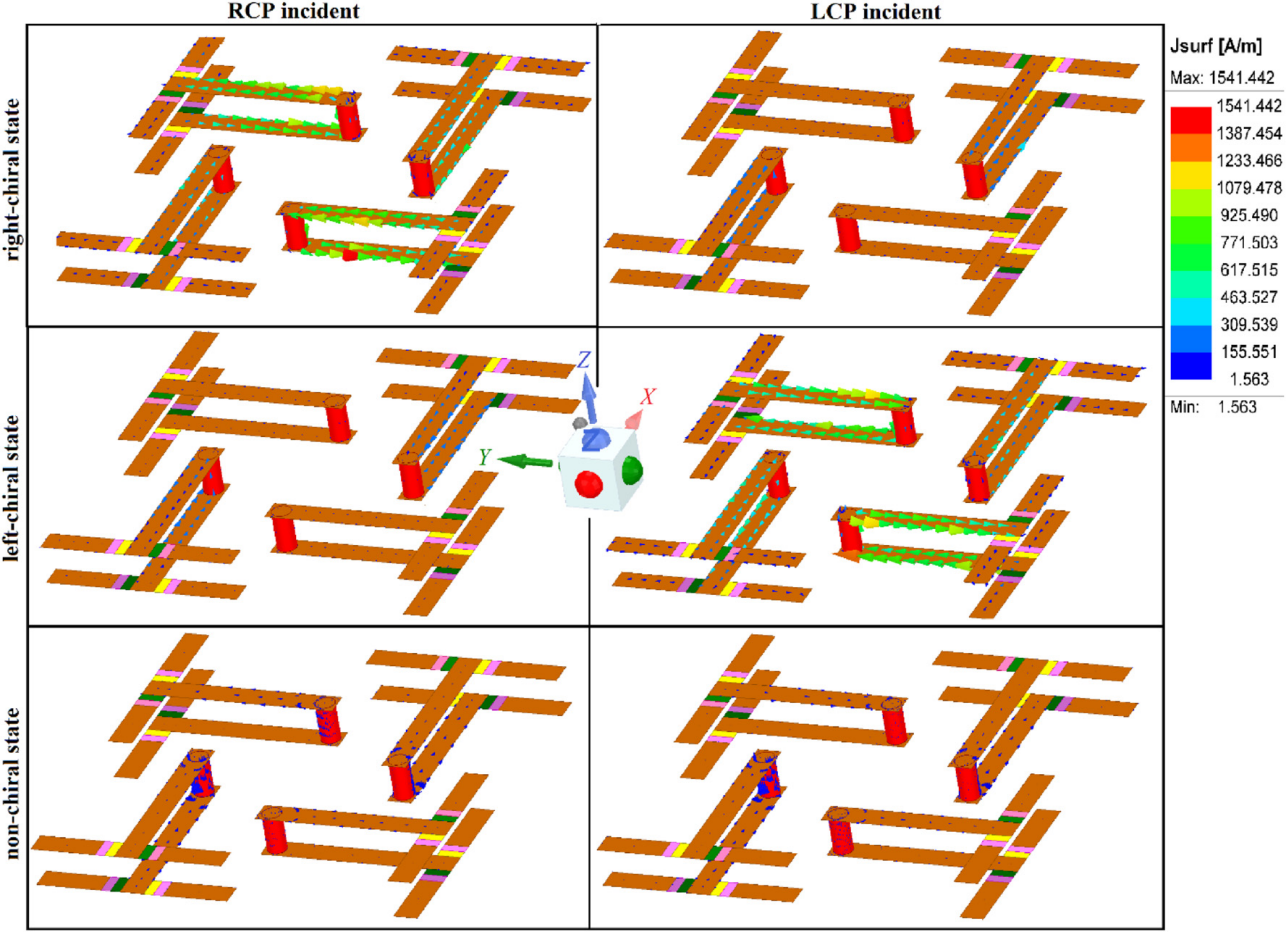
Surface current distribution for right-chiral, left-chiral and non-chiral states for RCP and LCP incidence at 9.3 GHz.

Surface current distribution for different states is presented in Figure 5 for both RCP and LCP normally incident waves at 9.3 GHz at the same phase. It can be observed from Figure 5 that the RC state interacts maximally with RCP light which induces surface current in the meta-molecule while it remains inactive for LCP light. Similarly, the LC state responds maximally to LCP light which induces maximum surface current while the RCP light excites a negligible surface current and is passed unnoticed. It is important to note that the surface currents shown in Figure 5 are for a particular phase of the incident field due to which the predominant currents are only on the diagonal meta-atoms with substantially small currents on the off-diagonal meta-atoms. However, for different phases, these currents can appear on off-diagonal elements. Similarly, there can be phases at which surface currents appear both on the diagonal as well as the off-diagonal meta-atoms. As the NC-state does not interact with both spins of light, the induced surface currents are negligible.

**Figure 6:**
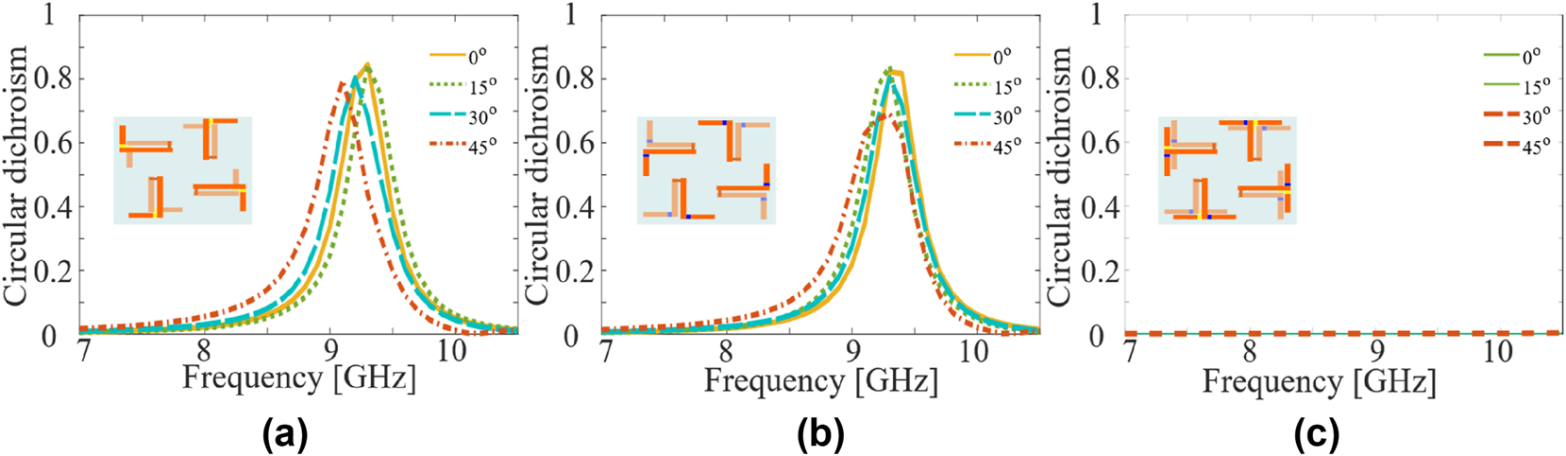
Variation of the circular dichroism with incidence angle for (a) the RC state, (b) the LC state, (c) the non-chiral state.

As in most practical applications, the EM wave can impinge from an arbitrary incidence angle, it is therefore important to investigate the chiroptical response of the proposed metasurface under obliquely incidence. In this regard, we simulated the metasurface under oblique incidence from 0° to 45° and present the results in Figure 6 for all three configurations. It can be seen that the CD is quite robust against variation in the incidence angles for all left-chiral, right-chiral and non-chiral states as presented in Figure 6(a)–(c), respectively. We observe that CD decreases for the LC case in Figure 6(b), however, it remains above 60 % for incidence angles up to 45°. We find that the sub-wavelength size of the meta-molecule (0.322*λ*_o_), judicially positioned vias and parametrically optimized dimensions of the meta-molecule contribute to the robustness against the variations in the incidence angle. This agrees well with previous findings on non-chiral and non-reconfigurable chiral meta-molecules [2], [38].

## 4 Experimental verification

**Figure 7:**
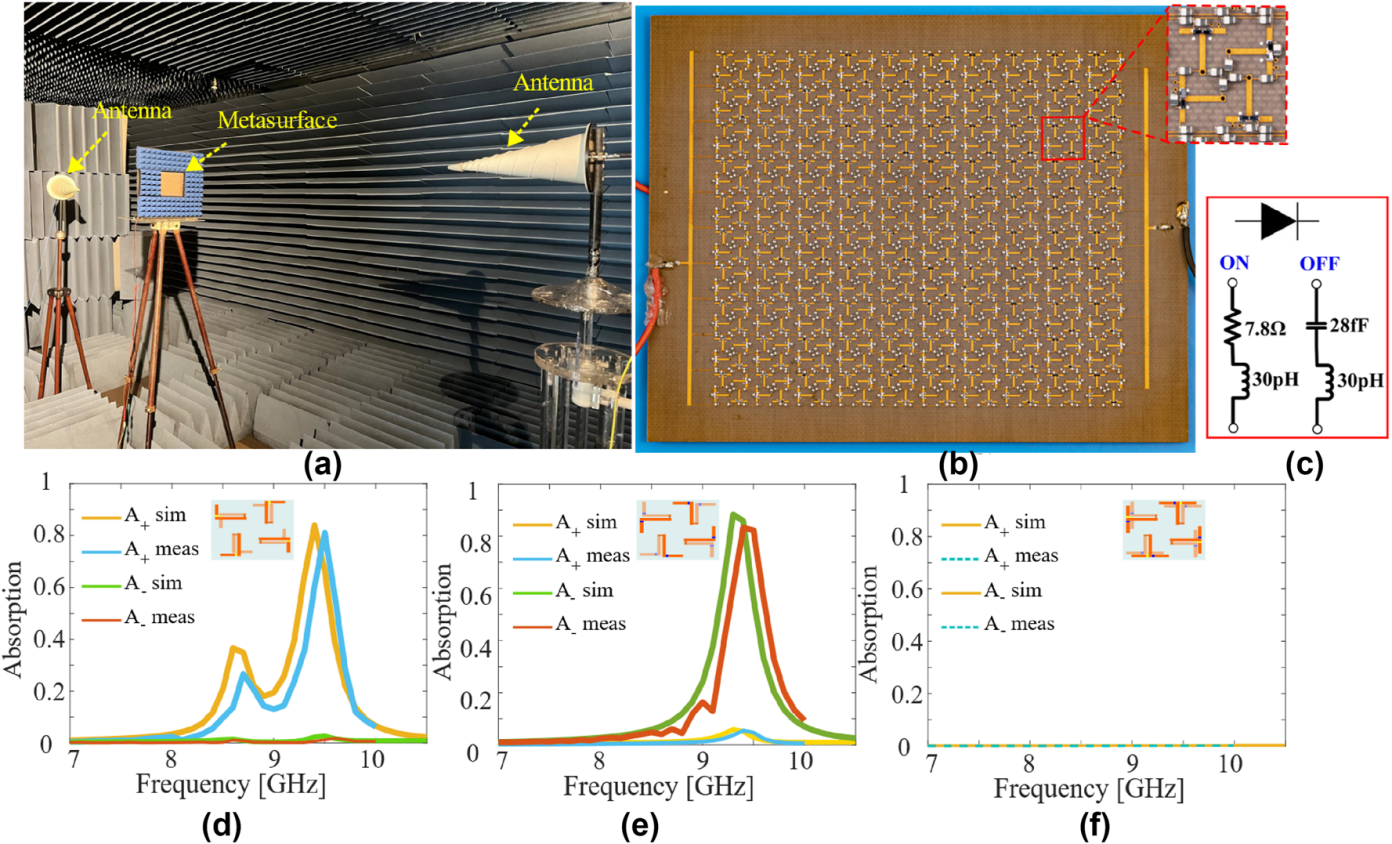
Metasurface fabrication and absorption spectrum measurement. (a) Measurement setup. (b) Top view of the fabricated metasurface and a zoomed view of the metasurface where biasing lines, lumped elements (PIN diodes and inductors) are visible. (c) Equivalent circuit for the ON-state and OFF-state of the PIN diode. Measured and simulated absorption for RCP and LCP incidence for (d) the RC state, (e) the LC state and (f) the non-chiral state.

We fabricate and characterize the reconfigurable chiral metasurface to experimentally demonstrate its spin-selective absorption characteristics. Figure 7(b) shows the top layer of prototype (the bottom layer has an identical meta-molecule distribution as can be seen from Figure 1, but has slightly different placement of bias lines). We fabricate the metasurface on a 120 × 120 mm^2^ F4B substrate using established printed circuit board etching and component soldering technology. The fabricated design is composed of 10 × 10 unit-cells where each unit cell has top and bottom metallic layers which are connected through metallic vias. The bias voltages for the PIN diodes are supplied by bias lines. In order to minimize the effects of bias lines on the functionality of the proposed structure, the biasing lines are taken with minimum thickness so that coupling with incident fields is minimized. Moreover, lumped inductors are judiciously positioned in the bias lines so that they block the high-frequency alternating currents and allow the biasing dc currents. Furthermore, the overall topology of the bias lines is kept such that it they are symmetric enough so that one polarization is not preferred over the other so that the original functionality of the metasurface is not significantly disturbed. In spite of all this, it will be seen that the inclusion of the bias lines slightly affects the performance of the metasurface but allows it to retain its salient properties.

For measuring transmission coefficients, the fabricated prototype is characterized inside an anechoic chamber. As shown in Figure 7(a), the metasurface is placed between two circularly polarized conical horns (EMCO 3102) operating from 7 to 10 GHz. One antenna transmits circularly polarized wave while the other receives the transmitted wave after passing through metasurface. For co-polarized transmission coefficients, transmitting and receiving antennas have the same helicity while for cross-polarized coefficients, the helicity of the receive antenna is reversed. The Keysight E8363C Vector network analyzer (VNA) is connected to the transmit and receive antennas to co-ordinate the measurement.

Different states of the metasurface are obtained by forward biasing the corresponding PIN diodes while reverse biasing others through DC source. Equivalent circuits of the PIN diode in the ON and OFF-state is shown in Figure 7(c). Both co- and cross-polarized transmission and reflection coefficients are measured for all the three states, from which the corresponding absorptions for RCP and LCP waves are calculated using equations (2)–(5). Figure 7(d)–(f) show the measured and simulated absorption coefficients for the two enantiomeric states and the non-chiral state of the structure. It can be seen from Figure 7 that the measured and simulated results are in good agreement with each other. It is important to note that the simulation and measurement results shown in Figure 7(d)–(f) are for the metasurface in the presence of bias lines. The geometric arrangement of the bias lines weakens the enantiomeric relation between the RC-state and LC-state as is evident from the simulation and experimental absorption spectra of the RC and LC-state in Figure 7(d)–(e). We observe that the RC-state has an extra smaller peak around 8.6 GHz. Nevertheless, we see that the main functionality of strong CDs of ±0.8 is achieved for the LC and RC states at the operating frequency for both simulation and experiment. The experimental resonance is slightly shifted up in frequency, most likely due to slight substrate property deviations and/or fabrication errors, but such frequency shift can be easily corrected through fine adjustments in meta-atom dimensions. At the non-chiral state, the cell is transparent and high-efficiency transmission is achieved.

**Figure 8:**
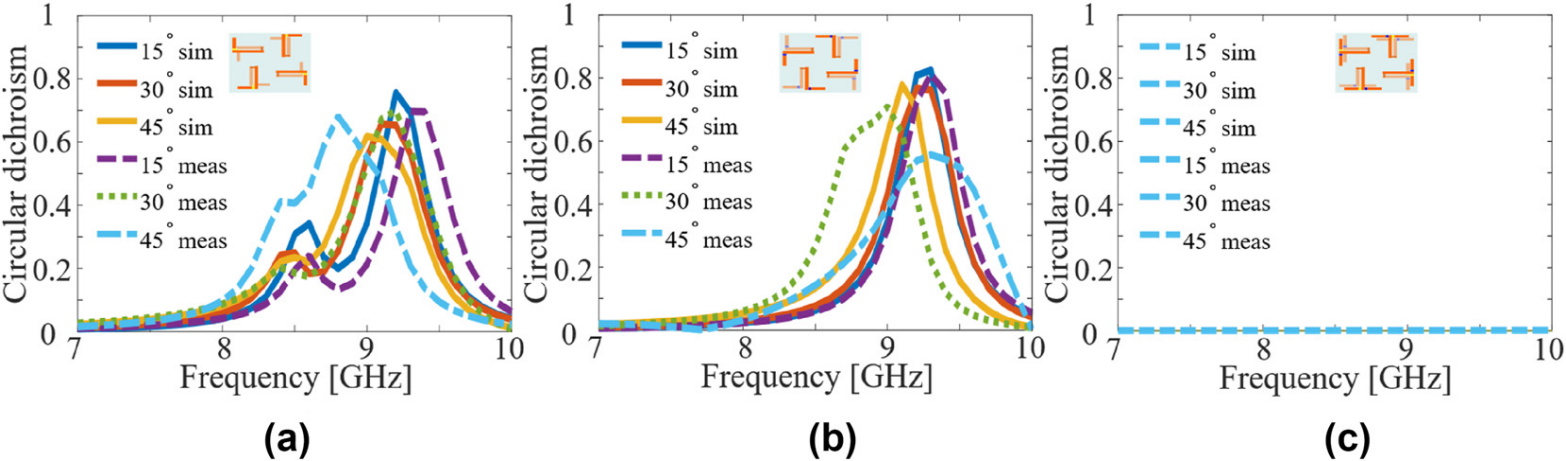
Comparison of simulation and experimental magnitude of circular dichroism (|CD|) for oblique incidence. (a) RC state, (b) LC state, (c) non-chiral state. The insets show the meta-molecule schematic for the respective states.

Figure 8 shows a comparison of the circular dichroism of the proposed design (in the presence of bias lines) under oblique incidence. It can be observed that CD remains within an acceptable range for all three states. The discrepancy between the simulation and experimental results can be attributed to the small size of the metasurface, the geometrical asymmetry caused by the presence of bias lines and fabrication imperfections.

## 5 Conclusion

We have designed and experimentally realized an ultrathin electronically reconfigurable chiral metasurface which exploits the intrinsic symmetries of the meta-molecule to achieve a tunable and operating-band invariant spin-selective absorption of light. We demonstrated that based on the chosen state (RC, LC or non-chiral) of the meta-molecule, the metasurface can either absorb RCP light only (RC state), absorb LCP light only (LC state), or transmit both spins with high efficiency (non-chiral state), at the same frequency of 9.5 GHz. This is achieved using a novel meta-molecule composed of four T-shaped meta-atoms. By electronically reconfiguring the PIN-diodes, we created two enantiomeric states (the LC and RC states) and a non-chiral non-resonant state of operation. In the chiral states, the CD exceeds 82 % over a 3 % operating band centered at 9.5 GHz for both enantiomeric states. We also showed that the circular dichroism remains robust to the variations in the incidence angle up to 45°. We have performed experimental demonstration and obtained results which agree very well with our simulation results. The reconfigurable chiral metasurface hereby proposed is hence a prominent candidate for a wide range of applications in imaging, wireless communication and medicine.
